# Physical Fitness and Motor Competence in Chinese and German Elementary School Children in Relation to Different Physical Activity Settings

**DOI:** 10.3390/children8050391

**Published:** 2021-05-14

**Authors:** Andreas Hohmann, Xinchi Yuan, Manfred Schmitt, Hui Zhang, Micha Pietzonka, Maximilian Siener

**Affiliations:** 1 Institute of Sport Science, University of Bayreuth, Universitätsstraße 30, 95447 Bayreuth, Germany; Andreas.Hohmann@uni-bayreuth.de (A.H.); Xinchi.Yuan@uni-bayreuth.de (X.Y.); Micha.Pietzonka@uni-bayreuth.de (M.P.); 2 Department of Psychology, University of Koblenz-Landau, Fortstraße 7, 76829 Landau in der Pfalz, Germany; SchmittM@uni-landau.de; 3 Department of Physical Education and Training, Shanghai University of Sport, 650 Qingyuan Ring Rd, Yangpu District, Shanghai 200438, China; zhang_hui@zju.edu.cn; 4 Department of Training Science, Zhejiang University, 866 Yuhangtang Rd, Xihu, Hangzhou 310028, China

**Keywords:** intercultural comparison, motor development, physical activity, mediation analysis, school sport, club sport, family sport, outside play

## Abstract

(1) Background: Children with greater physical activity (PA) may show a higher physical fitness (PF) and motor competence (MC) compared to peers with less PA. The purpose of this study was to examine the relationship between moderate-to-vigorous physical activity (MVPA), PF, and MC in 8- to 9-year old children in Germany and China. MVPA was differentiated into five PA settings: family sport, club training, school sport, leisure sport, and outside play. (2) Methods: This longitudinal study comprised *N* = 577 children (*n* = 311 girls, *n* = 266 boys) who were studied over a one-year period. Each child’s PF and MC was determined using sports motor tests. The children’s PAs were measured using a questionnaire. (3) Results: The children’s PA was positively associated with PF and MC. The MVPA-settings: family sport, leisure sport, outside play, school, and club sport, explained between 18 and 23 percent of the variance in selected PF and MC characteristics in a multivariate linear regression analysis. (4) Conclusions: An increase in the children’s MVPA might be an appropriate aim in the school sport in Germany as well as in the club sport system in China. Furthermore, family sport should be enhanced in Germany and outside play activities in China, respectively.

## 1. Introduction

Physical fitness (PF) can be defined as the overall performance in various strength, speed, and endurance tasks in a specified physical, social, and psychological environment [1]. Tests for the assessment of PF are an integral part of many general test batteries such as, e.g., the German Motor Test 6–18 [2]. Motor competence (MC) summarizes the degree of proficiency in a wide range of motor tasks, as well as the movement quality, coordination, and control leading to a particular motor performance [3,4]. PF as well as MC are key factors of physical activity (PA) and of physical, psychological, and social health and well-being in short and long-term development [5,6,7]. For example, in their conceptual model, Stodden et al. [8] identified MC and the mediators PF and perceived MC as key factors enhancing or lowering the PA levels of children and, thus, as an effective mechanism influencing the risk of obesity and healthy development of children. Their model proposed not only synergistic relationships among MC, perceived MC, health-related PF, and obesity with relations to strengthen over time but also a feedback loop from PA back to MC and PF, which supports our assumption that children with greater physical activity (PA) may show a higher physical fitness (PF) and motor competence (MC). Evidence indicates that PF and MC is effective in preventing obesity and excess weight [9,10,11], which is increasingly important due to a growing number of children and adolescents being overweight or obese. Moreover, PF and MC are positively associated with long-term PA [5,12].

Although one might assume that any level of children’s PA is better than a sedentary lifestyle, our study focused on moderate-to-vigorous physical activity (MVPA; [13]), as strong evidence exists that MVPA is not only positively associated with PF in children [14,15] but also an increase of the intensity from light-intensity to moderate-to-vigorous or even to vigorous PA is important to improve their PF [16,17,18] and body composition [19,20]. Furthermore, an increase of objectively measured time in MVPA reduces infant weight gain and diminishes the risk of adolescent obesity [21,22].

In regard to the association between PA, PF, and mental and cognitive health outcomes, the 2020 guidelines of the World Health Organization [23] stress for the age group of children at the age of 5–17 years not only an average of 60 min per day of MVPA based on mostly aerobic workload, but also the incorporation of vigorous PA on at least 3 days a week to strengthen muscles and bones. In addition to that, the WHO recommends limiting the times of sedentary behavior, and especially of recreational daily screen time, although no precise threshold or daily pattern is formulated.

Today, in China, many factors restrict children from meeting the WHO [13] recommendations of 60 min MVPA per day. From a school perspective, there are many problems that prevent children from attaining physical education [24]. These problems range from deficits in physical education knowledge concepts to poor sports equipment and facilities [24], lack of time for MVPA after school due to a large amount of time needed for additional study and homework thereafter [25,26,27]. From a family perspective, the lack of MVPA stems from improper educative concepts and educational styles, as well as the side effects of the one-child policy, which has led to a fear of sports injuries in parents and grandparents [28]. In addition to that, from an individual perspective, the lack of MVPA also results from environmental constraints such as restricted access to specialized venues, such as swimming pools, tennis courts, skiing areas, etc. [29,30]. Furthermore, in Germany, where the majority of elementary school children participate in club sports and the national guidelines recommend a daily MVPA of on average 90 min per day [31], MVPA declines as children get older. Whereas at the age of around 7 years, at least the male first graders fully, and the girls to a great extent, meet the WHO [13] recommendations of 60 min MVPA per day, in the following age groups many children are already less physically active than recommended [32], with a declining trend in the girls from 2009 to 2017 [33]. Furthermore, the high percentage of club membership with 80.9% of the boys and 61% of the girls in elementary school age also decreases slightly with rising age [34].

To date, different studies on children indicate that MVPA is influenced by multifaceted aspects, including social, biological, behavioral, psychological, and environmental factors [32]. Amongst the factors that are negatively associated with MVPA deficits in school children, the environmental barriers towards outside play activities play an important role [35,36]. Furthermore, a low quantity and quality of family sport [37,38,39,40], school sport [41,42], informal outside play [43], and the lack of institutionalized early sport club training might also lead to a lower MC degree [44,45,46,47]. Poor MC can, in turn, lead to a motor skill “proficiency barrier” [48] that impairs future sport participation [49], as the deficits in necessary motor skills hinder individuals from taking part in sports, games, and other context-specific and skillful activities [48]. Additionally, children and adolescents with poorer MC and PF may be more likely to lead a sedentary lifestyle in adulthood [50], whereas early MC [51], as well as adolescents’ participation in sport-related PA, impacts PA in adulthood positively [52]. In contrast, greater PF and MC in childhood may be predictive of later PA, as children with better PF and MC could find it easier to be physically active. Although PA components are relatively transitory, children with higher measures of PF and MC might be more likely to engage in PA as adolescents and adults compared with peers with poorer PF and MC. In addition to the fact that the initiation and maintenance of regular PA in adults depends on a multitude of biological and sociocultural variables [53], significant positive associations exist between PF, MC, and gross motor development [54]. These effects on and of MVPA may be different for children of the investigated nationalities in China and Germany due to social influences on sport participation and environmental factors that enhance or reduce MVPA.

Several attempts were made to compare the MVPA, PF, and MC of European children with American, African, Australian, and Asian children [55,56,57]. The comparisons between European and Chinese children (the Global Matrix 2.0 could not report specific data on Germany; [57]) revealed that overall MVPA is systematically higher in European countries. This result is mostly based on the substantially higher participation of European children in organized sport activities and active play. On the other hand, Chinese children exhibit slightly higher levels of family and school sport activities [57]. As could be expected, the lower overall MVPA in China, with less than 22% of the children meeting the WHO recommendations of 60 min/d [58], is associated with a low level of PF with only 3 out of 10 Chinese scholars achieving an excellent or good result in the national fitness standards [59]. Facing these research findings, a recent consensus statement of Chinese and international experts [60] called for governmental policies that tackle the deficits of Chinese children in MVPA and PF especially through a better availability of local community-based sport clubs to increase organized sport activities, as well as by improving the built environment (neighborhood design, parks and recreational facilities, walkable sideways, and bike lanes) as important resources for outdoor play activities.

Despite the global attempts to monitor PF in children of many countries with more than half of the World’s population, until today, to our knowledge, no common test standard has been established. The lack of uniform intercultural comparisons reduces the chances of more comprehensive insights into the causes of various PF and MC development constraints. This is particularly true in regard to the comparison of Chinese with German children. Moreover, there are important limitations to the existing studies in this field. Only a few of these studies have been able to fully consider the effects of different environmental factors. For example, in former intercultural studies, MVPA was not differentiated into participating in club sport, family sport, outside play, or all of these settings [61]. Additionally, different methods of data collection were used. Some previous studies, for instance, used questionnaires to assess MVPA but did not distinguish between frequency, intensity, type, and different settings. Similarly, there has been an absence of comprehensive and validated measures for a variety of PF and MC characteristics. Thus, in the studies that considered environmental factors, detailed diagnostic PF or MC profiles were not included [62,63,64]. Therefore, the purpose of this study was to examine the relationship between the physical activities and general PF and MC in 8- to 9-year-old children from two different countries.

The aim of this cross-cultural study was to investigate the culturally determined influence of MVPA on children’s PF and MC. Based on other cross-cultural studies of children in late childhood and adolescence [65], it can be assumed that Chinese and German children also differ in their weekly MVPA. To analyze these differences in MVPA in more detail, five different MVPA settings were considered: family sports, leisure sports, school sports, club sports, and outside play. It is to be expected that, in China, the majority of MVPA relates to school sports and family sport activities, while in Germany, however, club sports play a major role. The different designs of MVPA in the different settings then, in turn, affect PF and MC. In a first step, we, therefore, examined whether there were any differences at all in the level of PF and MC of children from both cultures. In a second step, the influence of the activity settings on the development of PF and MC was examined in more detail. The particular advance of this study on the influence of five different PA settings on children’s PF and MC is the novelty of combining a cross-cultural and longitudinal study design. Given the shortage of longitudinal studies on sophisticated effects of MVPA on PF and MC, and in order to obtain differentiated and nation-specific information on the yearly increase of PF and MC in this age group, a longitudinal study of one year was chosen as the study design, which also allows us to determine the magnitude and effect size of the change in the individual parameters.

## 2. Materials and Methods

### 2.1. Study Design

This study examines how culturally determined MVPA affects children’s PF and MC and whether differences between China and Germany can be identified in this regard. The entire longitudinal study took place over one year. The parents or guardians of each child were asked to complete at home an adapted short form of the German version [66] and the Chinese version of the International Physical Activity Questionnaire (IPAQ_SF; [67]). It is important to point out that, in contrast to the original IPAQ-SF, the parents were not only asked about the MVPA during the recent 7 days, but also about the average number of days per week their children participated in MVPA over the last 12 months. The questionnaires were administered to the parents of all *N* = 577 participants (*N_China_* = 296; *N_Germany_* = 281) at the beginning and end of the study to assess the weekly MVPA of the participants. 

Here, MVPA was differentiated into five different MVPA settings: (1) family sport, (2) leisure sport, (3) school sport, and (4) club sport sessions, as well as (5) outside play activities. Additionally, at the beginning and at the end of the study, the physical fitness and motor competence of all participants were tested using motor tests. A general linear model with repeated measurements (GLM) was used to identify differences between the two groups of participants (China and Germany) and a mediation analysis was conducted to evaluate the correlations between MVPA, and PF and MC, respectively.

### 2.2. Participants

A total of *N* = 577 2nd grade students (approximately 8–9 years old) participated in this study. Among these elementary school students, *n_CHN_* = 296 were children from China (*n*_Male_ = 145 and *n*_Female_ = 151) and *n_GER_* = 281 were from Germany (*n*_Male_ = 121 and *n*_Female_ = 160). The average age of all participants at beginning of the study was *M* = 7.79 (*SD* = 0.36; *Min* = 6.93 years; *Max* = 9.63 years; *T*_GER‒CHN_ = 1.95, *p* > 0.05). All of the children were healthy and free from diagnosed orthopedic and neurological impairments. No child showed a body mass index (BMI) above the 94th percentile (overweight; see [68]).

In Germany, children from eight elementary schools were included. They were located in a medium-sized county with a population of 216,093 inhabitants. In China, also, eight elementary schools were involved. They were located in the metropolitan area of Shanghai. The chosen district of Shanghai is predominantly composed of residential communities and has a population of 1.313 billion inhabitants. Both settings correspond to the aforementioned nation-specific living conditions that each were assumed to allow for comparably high PF and MC.

As part of the study, all participants and their parents were fully informed about the content of the testing and the resulting studies, and their written consent were obtained from each participant in both countries.

The study and research design were in line with the Declaration of Helsinki and was approved by both the ethics committee of the Shanghai University of Sport and the University of Bayreuth. Additionally, the responsible School Sport Administration of the Yangpu District of the Municipality of Shanghai (China), the District Office of the Fulda Region (Germany), the Sports Department of the Municipality of Fulda (Germany), as well as the State Office of School Education of Fulda, approved the study and research plan.

### 2.3. Physical Fitness and Motor Competence Tests

All participants were tested both at the beginning of the longitudinal study (Moment 1) and after one year at the end of the study (Moment 2) with a test battery consisting of two anthropometric tests (body weight and height), six physical fitness tests (sprinting, agility, arm and upper body strength, leg strength, and endurance performance), and three motor competence tests (coordination, balance, and ball throwing performance). According to the longitudinal pre-post study design, the two participant groups were tested in parallel. Each of the standardized tests was administered according to existing test protocols, which included a detailed description of the test items, the exact test setup, the demonstration of the test item, the administration of the test phases, and the measurements. Body weight was measured in kilograms to the nearest tenth using a digital scale (BF-350; Tanita, Arlington Heights, IL, USA) that was calibrated before each use against a standard weight. Body height was measured in centimeters to the nearest hundredth using a digital stadiometer (Digi-Kit, North Bend, WA, USA) that was also calibrated against a standard height.

#### 2.3.1. 20 m Sprint (PF)

Sprint performance was recorded using a 20 m sprint (~21.9 yards). On each of the two possible trials, subjects started 0.3 m in front of the starting line. Time was stopped using light gates (Brower Timing Systems; Draper, UT, USA). The test achieved an objectivity value of 0.86 and a reliability value of 0.96 [69].

#### 2.3.2. Standing Bend Forward (PF)

A standing torso bend forward was administered to test flexibility. Here, the participants attempted to reach as far as possible with their fingertips beyond their feet and to hold this position for at least 3 s [70]. The floor level was scored as 0 cm, and the distance of the fingers in cm to ground level was recorded. A low range above ground level was recorded as a negative distance, and anything below the floor level was recorded as a positive value. There were two attempts allowed, and the maximum value was determined by the best of two trials. The test achieved an objectivity of 0.99 and a reliability of 0.94 [71].

#### 2.3.3. Push-Ups (PF)

This test required participants to perform as many push-ups as possible over 40 s. To execute the test with a correct technique, the body movement started from the initial position lying down on the floor with the hands touching behind the back. Then, both hands were put down on the floor and extended. In this push-up position, one hand had to touch the other hand and then move back. A complete repetition was only evaluated when the upper body was laid down again on the mat and hands touched each other behind the back. Only one attempt was performed. Bös [71] rated objectivity as 0.98 and reliability as 0.69.

#### 2.3.4. Sit-Ups (PF)

Similar to the push-up test, the sit-up test also assessed the number of correctly performed sit-ups in 40 s [2]. After a short practice phase, only one test attempt was granted, and the number of correctly executed sit-ups was counted. To execute the test with a correct technique, the body movement started from the initial position lying fully on the back on the mat with the knees bent to 90 degrees and the fingers of each hand touching the head behind the ears. Then, the upper body had to be lifted up until both elbows touched the knees. Then, the upper body moved down again until the participant laid on their back again on the mat. The objectivity of this test was 0.92, and the reliability was 0.74 [72].

#### 2.3.5. Standing Long Jump (PF)

In the standing long jump test, the jump distance was measured in cm. Each subject had two trials, the better of which was recorded. A complete pause was observed between the two trials. The test achieved an objectivity of 0.99 and a reliability of 0.89 [2].

#### 2.3.6. Six Minute Endurance Run (PF)

In the endurance test, participants attempted to run as many laps as possible around a 9 × 18 m volleyball court in six minutes. The distance achieved was noted in meters. The test was performed by a total of 15 people at the same time. The objectivity of this test was 0.87, and the reliability was 0.92 [2].

#### 2.3.7. Ball Throw (MC)

In the ball throwing test, the throwing distance was measured in cm orthogonal to the line of throwing. Subjects threw an 80-gr ball from a standing position three times in succession. As in the previous tests, the best value was evaluated. The test could be evaluated in own studies (*n* = 1800) with a reliability of 0.77.

#### 2.3.8. Sideward Jumping (MC)

The number of two-legged jumps that a participant could perform within 15 s between two 50 cm × 50 cm (1 cm ≈ 0.4 inches) squares was measured [73]. Only jumps in which none of the boundary lines were touched were counted. A total of two trials were performed, with a pause of at least 2 min between each trial. The mean of both trials was used for further calculations. The objectivity of this test was 0.99, and the reliability was 0.89 [71].

#### 2.3.9. Balancing Backwards (MC)

Participants balanced backward on 6 cm, 4.5 cm, and 3 cm wide wooden beams [73]. Two trials were made on each beam and the number of steps (feet fully raised) were counted before exiting the beam. A maximum of eight steps/points could be obtained per beam, limiting the total maximum score for this test task to 48. The test received an objectivity of 0.99 and a reliability of 0.73 [69].

All tests were performed by qualified personnel during regular school hours (8 a.m.–12 p.m.), and a uniform warm-up was performed before the start. The 6-min endurance run was always the last test in the series of tests. All eight tests, besides the ball throw, were examined in a whole series of studies by various authors [69,71,72] with regard to the test standards. Bös [71] analyzed the test battery’s psychometric properties for a sample consisting of nearly 50,000 school children and adolescents. The validity of the three elementary balancing, jumping, and ball handling tests for motor competence (MC) as an underlying general motor ability in children aged 6–11 years was recently confirmed by Bardid et al. [74], as these authors found one-dimensionality of even 14 different motor test tasks of the Bruinink-Osseretsky-Test of motor proficiency (BOT-2).

### 2.4. Physical Activities

At the end of the study, parents of all *N* = 577 participants completed a paper format questionnaire that was based on the International Physical Activity Questionnaire [IPAQ; 67] in a short version and in combination with the Chinese format [75]. The paper-based questionnaire asked the parents about the average number of days per week their children participated in moderate-to-vigorous sports over the past 12 months. The questionnaire comprised five MVPA variables according to the different settings of (1) family sport, (2) leisure sport, (3) school sport, and (4) club sport sessions, as well as (5) outside play activities. In the “outside play” category, only days were recorded when children had played outside for more than 60 min [13]. All other forms of guided MVPA were expected to last longer than 45 min.

In the questionnaire, in addition to the days with MVPA, the exact number of hours of MVPA and any associated sports were also collected. However, it turned out that the respective time data strongly depended on the activity performed, and a comparison of the concrete times, thus, seemed difficult. For example, weight training and continuous running were performed for an average of 45 min, whereas hiking or horseback riding sometimes lasted 3–4 h. Moreover, since only in very rare cases was more than one activity of the same MVPA setting practiced in one day, this study was oriented towards the simpler consideration of days in which MVPA was practiced in the respective settings.

The validity and reliability of the parental adaptation of the Chinese short form of the IPAQ-SF [76] in regard to MVPA of 9-year old children was confirmed by Liu et al. [28]. In Germany, the direct applicability of the IPAQ-SF in youths was confirmed by Rütten et al. [66], although the reported test-retest reliability scores were rather low. In order to evaluate the quality of the questionnaire, it was given to the participants again after one year. As could be expected, Cronbachs’alpha was highest in organized club sport participation (α = 0.82). This quite regular activity was followed by outside play (α = 0.62), family sport (α = 0.52), and school sport (α = 0.51). Cronbachs’ alpha (α = 0.40) was lowest in the item leisure sport activities. In regard to validity, factor analysis showed one-dimensionality (*EV* = 1.62) with an explained variance of 32.36 percent for all five questions.

### 2.5. Data Analysis

For all analyses, the software *SPSS* (version 26; SPSS Inc., Chicago, IL, USA) was used. The significance level was set to *p* ≤ 0.05, and significant results were marked with * in the text. If an analysis was highly significant (*p* < 0.01), it was marked with **.

The sample size for the two groups in each country (85/85) was calculated according to Hopkins and Batterham [77], with an effect size of 0.6, an alpha risk of 5%, and a beta risk of 10%, and was surpassed in the present study in both sexes (121/145 in the boys, and 160/151 in the girls). In order to avoid a relative age effect, all data were separated for boys and girls and then a linear regression was calculated for each test item with age (in months) as independent variable [78,79]. The resulting residuals were saved as *z*-standardized residuals [80,81,82]. This resulted in standard deviation *z*-scores for the nine selected subtests, which were used to calculate the interplay between the five MVPA settings and the nine test performances. In order to ensure a better comparison of the sprint data, the z values were additionally multiplied by “−1”, thus, turning the better sprint results into positive z values.

Descriptive statistics were used to compute the mean values (M), standard deviations (SD), standard errors (SE), and Cohen’s d for the PF and MC components, and also for the weekly MVPA in the settings: (1) family sport, (2) leisure sport, (3) school sport, and (4) club sport sessions, as well as the number of weekly (5) outside play activities that lasted longer than 60 min [13].

General linear models with repeated measurements (GLM) were used to test for descriptive characteristic differences and the test (raw) values regarding the 2 × 2 × 2-factors time, nationality, and sex. Analysis of covariance was used to examine differences for each of the nine test items, while adjusting for child age in months.

In this case, if differences were found between the two nationalities, for boys and girls, nine separate mediation analyses on the basis of a multivariate linear regression analysis [83] were calculated. Only the *z*-values were used for mediation. In the eighteen regression analyses, the mediator “nationality”, together with the five MVPA settings, served as independent, and each of the single PF and MC components were used as the dependent variable. The aim was to examine the relationships between the MVPA in the different settings and the PF and MC outcome for each gender to elucidate the causal relationships between the *z*-standardized test performances of the children in the two countries on the basis of the natural direct and indirect effects of the nationality and the five settings on PF and MC.

Finally, we used two multivariate regression analyses to examine the effects of the five independent variables formed by the PA settings: (1) family sport, (2) leisure sport, (3) school sport, and (4) club sport sessions, as well as the number of weekly (5) outside play activities, on the dependent criterion variables overall PF, and overall MC, respectively. Both the overall PF and overall MC were formed by the summated z-scores of the six PF and the three MC tests.

## 3. Results

### 3.1. Physical Fitness and Motor Competence Tests

Six physical fitness tests and three motor skills tests were used to measure the participants’ athletic performance at the beginning (Moment 1) and at the end of the study (Moment 2). The raw score results of the two performance test times are presented in Table 1. Other than the bend forward test in Chinese boys and girls, all differences between Moment 1 and Moment 2 are significant (*p* < 0.05).

Between the Chinese and German scholars, age (*T* = 1.96; *p* > 0.05) and body height (BH: *T* = 1.78, *p* > 0.05) did not differ systematically, only in the subgroups of the genders as the Chinese girls were significantly taller (BH: *T* = 2.37, *p* < 0.05). More differences occurred in body weight (BW), as in both sexes, differences between the nationalities (boys: *T* = 3.39, *p* < 0.01; girls: T = 2.01, *p* < 0.05) could be found.

**Figure 1:**
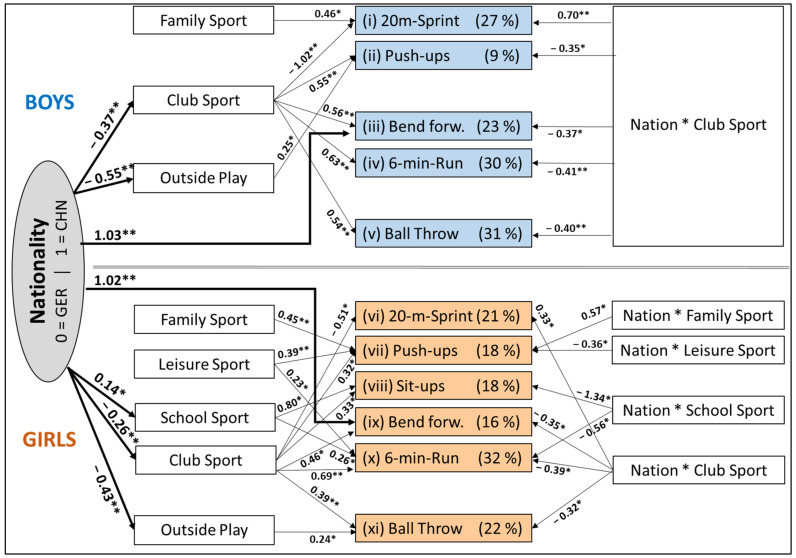
Summary of 11 (significant, * *p* ≤ 0.05, ** *p* ≤ 0.01) mediation analyses on the effects of the five MVPA settings family sport, leisure sport, school sport, club sport, and outside play on the PF and MC measures in Chinese and German boys (upper part) and girls (lower part).

The German children were better than Chinese children of the same age in the PF test items 20 m sprint, sit-ups, push-ups, and endurance run. In the 20 m sprint, the univariate main effect showed that German schoolchildren (*F_Nationality_* = 76.91; *df* = 1; 564; *p* < 0.05; *d* = 0.68) ran faster than the Chinese children (Table 1). Furthermore, boys generally showed better performances than the girls (*F_Gender_* = 19.99; *df* = 1; 564; *p* < 0.05; *d* = 0.31). In the push-ups test, the results were quite similar to those obtained in the sit-up test, with better performances in the total group of the German children compared to the Chinese group (*F_Nationality_* = 37.96; *df* = 1; 565; *p* < 0.05) and better performances obtained in the boys compared to the girls (*F_Gender_* = 7.92; *df* = 1; 565; *p* < 0.05). Additionally, In the sit-ups test, the German children showed better results than the Chinese pupils (*F_Nationality_* = 83.41; *df* = 1; 565; *p* < 0.05; *d* = 0.07). Generally, boys were also significantly better than the girls (*F_Gender_* = 7.40; *df* = 1; 565; *p* < 0.05). Although the German children reached more repetitions than the Chinese children in both testing years, this significant univariate effect in the second test (Moment 2) was reduced by the multivariate interaction effect of a systematically higher performance gain of the Chinese children during the following year up to grade three (*F_Time x Nationality_* = 18.58; *df* = 1; 565; *p* < 0.05). In the 6-min-run (Figure 1), the German elementary school children exhibited not only the greatest relative advantage compared to the Chinese group (*F_Nationality_* = 125.63; *df* = 1; 540; *p* < 0.05), but as a multivariate interaction effect, also a better performance development (*F_Time x Nationality_* = 8.98; *df* = 1; 540; *p* < 0.05). Thus, a difference of more than 80 m occurred in both genders. In addition to that, the GLM attested another multivariate interaction effect in that the German boys showed a better running endurance development from grades two to three than the Chinese children (*F_Time × Nationality × Gender_* = 5.48; *df* = 1; 540; *p* < 0.05).

Children from China were superior to children from Germany in the test items standing long jump and bend forward. The performance development in the standing long jump in Chinese boys and girls was quite similar over the time span of one year. Although the boys of both nationalities demonstrated higher performances than the girls (Table 1), none of the differences between the investigated groups were significant. In the bend forward test, the Chinese children performed much better than their German counterparts. The significant univariate effect in the differences between the two nationalities, especially in the second grade, was extremely high. Although the Chinese children stagnated and German children caught up a little in the following year (*F_Nationality_* = 202.78; *df* = 1; 563; *p* < 0.05; *d* = 1.03), the better flexibility of the Chinese children persisted. In addition, the girls turned out to be more flexible than the boys (*F_Gender_* = 7.92; *df* = 1; 565; *p* < 0.05).

The development rates (delta) were calculated on the basis of the raw scores over the one-year period and were used for the GLM analysis of the interaction effects in all group comparisons. The results of the one-year development of the six PF tests and three MC test are shown in Table 2.

In the areas of MC, German children were better than Chinese children in the two test items balancing and ball throw. In the balancing backward test, univariate main effects showed that the German children reached better results than their Chinese counterparts (*F_Nationality_* = 28.49; *df* = 1, 565; *p* < 0.05; *d* = 0.43), and the girls performed better than boys (*F_Gender_* = 44.31; *df* = 1; 565; *p* = 0.05; *d* = 0.47). Nevertheless, there was no significant interaction between time, nationality, and gender. In the test sideward jumping, the GLM documented a significant difference in gender development (*F_Gender_* = 4.29; *df* = 1; 565; *p* < 0.05; *d* = 0.25), which was probably caused mainly by the Chinese girls (Table 2). For example, the 8- to 9-year old Chinese girls showed an enormous increase in performance from Moment 1 to Moment 2 and surpassed the German girls, who developed much slower. Accordingly, the GLM confirms the significance of this multivariate interaction effect (*F_Time_*_x Gender_ = 12.02; *df* = 1; 565; *p* < 0.05), as well as the systematic difference in the development between the two genders in the two countries (*F_Time × Nationality × Gender_* = 21.77; *df* = 1; 565; *p* < 0.05). The ball throw measures the speed, strength and, besides that, the important ball handling skill of throwing. In this more specific MC test, the German children reached a performance development that was much better than that of their Chinese counterparts, especially in the boys, as they developed systematically better than the girls (*F_Gender_* = 4.21; *df* = 1; 564; *p* < 0.05). The GLM shows a multivariate interaction effect as the differences between the two nationalities grow, starting in the initial testing in the second class and continuing in the following one-year performance development (*F_Time x Nationality_* = 13.88; *df* = 1; 564; *p* < 0.05). Overall, boys generally performed better than girls in the areas of speed, power, core strength, throwing power, and endurance. Girls, on the other hand, had a clear advantage in flexibility and balancing.

### 3.2. Physical Activities

The questionnaire looked at average weekly days with MVPA. Comparing the results of both countries, it was noticeable that family sports, school sports and leisure sports were of more or less equal importance in both countries. MVPA in the school setting was practiced on an average of 2.5 days per week. Chinese children had minimally more days with school sport-related activity than German children. However, the differences for boys were not significant (*p* > 0.05). However, a significant difference could be detected for girls in this category (*p* = 0.02). Family sports and leisure sports were mostly performed less than once a week (0.66–1.0 days). In both categories, no significant difference between the two countries could be detected. In the category of club sports, German children achieved significantly higher values than Chinese children. While children in Germany participated in club sports once a week on average, children in China did so only every two weeks. The difference was even more pronounced in the MVPA setting “outside play activities”. Here, children from Germany were active 3–4 days a week. Children from China were only active half of the time (1.5–1.6 days). Looking at the differences between boys and girls, it was noticeable that German boys were significantly more active in the outside play category than girls. In all other MVPA settings, boys and girls performed similarly. The means (M) and standard deviations (SD) of the MVPA in the five different settings reported by the parents are presented in Table 3.

### 3.3. Mediation Analysis of the Relation between Moderate-to-Vigorous Physical Activity, Physical Fitness, and Motor Competence

The parental report data on the five PA settings were used for the mediation analysis to explain the effects on each of the six PF and three MC components separately for the boys and girls from the two nations. Eleven of the totally 18 analyses led to significant regression models (*p* < 0.05), which elucidated the relevance of the five MVPA settings and the mediator nationality on the respective PF and MC component. Figure 1 provides a comprehensive overview of the results where the five models obtained in the boys are shown in the upper part, and the six models of the girls in the lower part.

The mediation analysis in boys (Figure 1; upper part) shows that in Germany, in comparison to China, the far most influential setting was the club sport, which was crucial to five of the six investigated PF and to one of the three MC components. In addition, the German children showed a higher number of outdoor activities (Figure 1), which were positive for the push-up performance in the boys and the ball throw performance in the girls. The school sport was influential, especially in the girls of both nations. A higher amount of family sport was associated with slower sprint times in boys, but higher push-up performance, especially in the Chinese girls.

In regard to the single PF tests, the mediation analysis documented that the high impact of club sport on 20 m-sprint performance was moderated by nationality (*Beta_Nation × Club Sport_* = 0.70; *p* < 0.05). In the girls, the findings in regard to sprint running performance were quite similar. With 21% of the variance explained, the mediation analysis showed that club sport was also the primary factor for performance development for the girls. Furthermore, the running speed of the girls was largely determined by the higher efficacy of club sport participation in Germany (*Beta*_Nation × *Club Sport*_ = 0.33; *p* < 0.05).

The mediation analysis showed that the push-up performance of the Chinese girls depended to a higher degree on family sport, whereas the push-up performance in the German boys was primarily based on club sport training. In regard to the sit-up performance in the boys, there was no significant pathway from the MVPA in any of the five settings. In the girls, however, the situation was different, because 18% of the variance in the performance outcome could be explained by the five influence factors (Figure 1; lower part). The mediation analysis confirmed not only club and school sport activities as general factors of performance, but also that the advantage of the German girls in core strength was due to the higher impact of school sport in Germany. The strong interaction of nationality and school sports on the sit-up performance of the Chinese girls was an intriguing finding that showed the high relevance of physical education for the core strength performance of the Chinese girls.

In Figure 1, a striking mediation effect of the variable nationality on the performance in the bend forward flexibility test can be seen. Therefore, the torso and hip flexibility, both in boys and girls, not only depended on the volume of club sport activities but in part stemmed also from national differences (Figure 1, upper part). Therefore, in our study, the greatest differences between the Chinese and German children occurred in this component of the MC, in addition to biological factors, although club sport contributed, at least in German children, to body flexibility.

In the endurance test 6-min-run, the 8- to 9-year old German elementary school children of both genders showed the greatest relative advantage compared to their Chinese counterparts. In the mediation analysis of the 6-min-run, 30% of the overall variance in the boys (Figure 1, upper part) and 32% in the girls (Figure 1, lower part) could be explained by the five basic sport activity variables. In the set of environmental constraints, which played an important role in the development of running endurance, at least in the German boys, the highest impact on German boys and girls came from the club and school sport settings, suggesting that MVPA is an integral goal in such settings. Both parts of the figure illustrate that the better running endurance of the German boys and girls was, once again, primarily due to their club sport participation. In addition to that, in the girls, the running endurance also depended on the volume of school sport activities, which are less effective in the Chinese females.

The MC components balancing backward and sideward jumping of the total group were not significantly affected by the number of weekly sessions spent in the different sport settings. In regard to the ball throw, the mediation analysis showed that the performance of the boys primarily went back to the volume of club sport activities, which are more important to the German boys (see Figure 1, upper part). In addition to club sport, the ball throw performance of the girls of both nationalities was also based on outside play activities.

For a comparison with the existing knowledge on the interrelation between PA, PF, and MC, we calculated two multivariate regression analyses to clarify the influence of the five PA settings on the overall PF the German and Chinese children reached at the end of the investigation (moment 2) and another two analyses to elucidate the influence of the PA settings on the overall MC. Three out of the four regression analyses yielded significant results, as the model in regard to MC in the Chinese children did not reach significance (*F*_5;217_ = 2.12, *p* = 0.064).

The regression model of the overall PF of the Chinese children (*F*_5;217_ = 7.15, *p* < 0.001) explains 12.6% of the variance of the dependent variable PF, which is significantly influenced by leisure sport (*Beta* = 0.16, *p* < 0.05), as well as by club sport activities (*Beta* = 0.32, *p* < 0.001). In the regression model of the German participants (*F*_5;217_ = 7.15, *p* < 0.001), the determination of the overall PF is 12.4%. Here, the only significant predictor again is the club sport setting (*Beta* = 0.39, *p* < 0.001) as no other PA setting reached significance. Finally, the overall MC of the German children is also primarily determined by the PA executed in the club sport setting (*Beta* = 0.26, *p* < 0.01), but the outside play activities show at least a tendency towards significance (*Beta* = 0.13, *p* < 0.10).

In regard to the independent variables, family sport and school sport, there was no systematic effect either on the overall PF or on the overall MC in the children of both countries.

## 4. Discussion

The aim of this study was to examine the development and correlated factors of PF and MC of 8- to 9-year old Chinese and German elementary school children with regard to different MVPA settings. The present findings support the effectiveness of sport participation in addition to the obligatory school sports and expanded previous research in several important ways. In particular, the design of the study allowed for a more complete picture of the longitudinal mid-term influence of the different MVPA settings on PF and MC and the different effects of the typical urban environments and social conditions in China and Germany. As physical and motor development in children is influenced by various sport and play activities [84], the investigation of the relationship between the participation in different MVPA settings and the resulting PF and MC in the 8- to 9-year old scholars was focused on the following settings: family sport, leisure sport, school, and club sport activities as well as outside play activities. Generally, the results of the study indicated that most of the PF and MC components were positively associated with the amount of MVPA in the five settings investigated. The set of the investigated five different activity settings explained between 3–32% of the total variance in the nine different physical and motor characteristics in boys and girls, after adjusting for monthly child age. Although these associations between MVPA and the various physical and motor characteristics are only weak to moderate, the positive feedback loop between MVPA, PF, and MC introduced by Stodden and coworkers [8], in their developmental model of the influence of MC and PF on PA (and health), was clearly corroborated, as in the children from both countries, a higher amount of MVPA in the five settings was associated with higher PF and MC [5,85].

In terms of single PF and MC characteristics, we found that the more physically active the children were, the better they performed the tasks of running speed and running endurance, and the faster and further they jumped. Boys at the age of 8 to 9 years showed significantly faster running sprint and better jump abilities, had more strength and better running endurance, and reached a longer distance in an 80-g ball throw compared to girls. These findings support the frequently observed gender differences and are in line with previous research [86,87,88] that supports gender differences, especially in condition-based PF tests, with boys’ performance levels in strength, running speed, and jumping power tasks exceeding that of girls. Such gender differences in particular PF tests apparently exist in Germany as well as in China. In addition to a possible slight onset of a biological influence on the PF and MC development of the investigated 8–9-year old boys and girls, our results show that, especially, the higher amount of moderate-to-vigorous club sport and outside play activities in the German boys compared to the girls, as well as the higher amount of family sport of the Chinese boys compared to the Chinese girls contribute to the observed gender differences in PF and MC in both countries. In regard to the Chinese children, this finding is surprising, as Zhu et al. [58] in their nation-wide survey not only failed to detect such gender differences in the overall PF of Chinese children but even found a higher risk in the boys not to meet the age-related fitness standards.

In terms of single MC characteristics, we found that the more physically active children performed better in balancing backwards, sideward jumping, and the ball throw, which is in line with the general MC development trajectories reported by [89] for this age group. On the other hand, girls in both countries performed better than boys in the tests requiring a high MC, as represented by balancing backward and sideward jumping, which is in line with the nation-wide findings of Zhu et al. [58] in China. This advantage of the girls explicitly does not occur in the throwing performance, which is in line with the findings of Luz et al. [90]. The gender differences with regard to MC not only refer to locomotor skills but also to object control, such as ball handling, and can be explained by environmental influences (such as in Fig. 1 club sport and outside play activities) or their interactions with biological factors beginning already at preschool age [91,92]. In 8- to 9-year old elementary school children, the amount of MVPA in different environmental surroundings were likely to explain some of the gender differences and the speed of skill development in MC. At elementary school age, the type of sports and games that boys and girls execute in club and school settings also provide opportunities to practice and refine MC and particular skills, which may contribute not only to the gender, but also to differences between the nationalities [92,93,94]. For example, in Germany, children prefer throwing and running games, such as handball and soccer, which are more popular amongst boys than girls and, thus, may lead to higher ball throw proficiency [95]. In the mediation analysis of the underlying factors of the ball throw in the boys, it is important to mention that the variance explained amounts to 31%, one of the highest values in comparison with the other components of the PF and MC of the children studied. One possible reason is the dominant role of the diversification of the exercise contents in the age groups’ skill training delivered by the sport clubs [96]. The broad variety in the club sport exercises, aiming at a multifaceted skill acquisition be it based on a general sampling [97] or on a specialized sampling concept [98], and not only focusing on the particular techniques of the respective main sport is very effective at a young age, especially in the German age group population.

The mediation models provided insights into the various settings that enhance MVPA as a determinant of PF and MC in young children. In all five settings, an increase in MVPA can be beneficial for PF and MC, as well as for general health status [60]. It is well known from the literature that parents’ attitudes and parental support play an important role in MVPA, PF, and MC of children of elementary school age [99], although the positive influence of the mother and father is different in nature [100]. Despite the fact that, in Germany, 66% of the parents participate together with their children in MVPA [101], our findings show that, in China, where club sport is less common than in Germany, family sport naturally plays a more important role for promoting PF and MC. Our results showed that the Chinese children’s performance in the strength test sit-up was significantly more influenced by participating in family sports activities than in Germany. As 8- to 9-year old German boys and girls performed much better in this test, the interaction of family sport and sit-up performance in China could be interpreted in a way that family sport in China secures at least a parallel performance development of the youngsters, although on a lower level.

Schmidt et al. [34] reported that German children play leisure sports outside almost every day and at least 4 days per week, respectively. This time can be spent in playgrounds or, for example, in parks. In large cities such as Shanghai, outdoor sports are mainly limited to parks, which provide ample space and opportunities for leisure sports and, thus, promote MVPA, recreation, and health in society [102]. Parks are often used in both China and Germany for jogging and walking. In Germany, children of the age group studied generally preferred various types of play and game sports, whereas in open public spaces in China, a greater variety of leisure sport activities exists, such as tai chi, wushu, dance, gymnastics, and meditation, as well as various individual game sports, such as table tennis and badminton.

The most common setting of MVPA influencing MC in both countries is regular school sport, which varies between two and three lessons per week in Germany, whereas in China, three lessons per week are standard. As school sport in Germany, as well as in China, is predominantly based on regular physical education (PE) lessons and not so much on additional extracurricular sport events or voluntary afternoon courses, its volume does not vary much between Germany and China. Thus, it is assumed that any effect comes primarily from the content and intensity level of PE activities. We found that, in the power- and endurance-based fitness tests, sit-ups, and running endurance, as well as in the more coordination-based skill ball throw, school sport in Germany had a higher impact on the performance in the third grade, whereas in other abilities, such as sideward jumping, with approaching significance in China, a greater performance was detected. In China, generally, school sport seemed to be more relevant for PF and MC. The efficacy of school sports in China was especially responsible for the interaction of nationality and school sport on the sit-up performance of the Chinese girls. Additionally, in the ball throw test, the lower performances of the girls also may indicate differences between Chinese and German sport education. Health-related physical education curricula, with effective teacher training and support, have the potential to provide children with much more MVPA than they have received in typical physical education lessons [103,104]. In summary, the present findings on the effects of school sport in Germany and China have implications for physical education practice, in the sense that they support the call by Chen et al. [60] for more intense and versatile school sport in China focusing explicitly on aerobic and muscle-strengthening MVPA, as well as they confirm Granacher et al. [105], who promote strength and balance training during physical education lessons also in Germany. Therefore, teachers and coaches in both nations are encouraged to use specific PF- and MC-directed training programs [106], as well as different sport disciplines in combination with a moderate-to-vigorous training intensity level, to support the PF and MC of scholars with different expertise levels. Additionally, specific types of sports facilitate the development of strength, speed, endurance, coordination, and flexibility. Future studies should also uncover which type, volume, and training intensity level of the club sport sessions are best suited to enhance not only PF but also MC in elementary school children. This can be done through surveying sport disciplines, such as soccer, that might have predominantly contributed to the increased running endurance level of the German children. The Chinese children may have had an increase in motor coordination and flexibility from sports such as tai chi or gymnastics.

Regarding the effects of the club sport activities, the results showed that the boys participating in club sport had far better results in most of the diagnosed MC components than the girls. Furthermore, our findings indicated that the children with the highest volume especially of club sport activity performed much better compared to children with lower MVPA levels. The children that participated in club sports showed higher PF and MC than the other children, which is in line with the findings of Howie et al. [61] and Vallence et al. [107] and particularly relevant in Germany [108]. As club sports are more prevalent in Germany, where 61.0% of girls and 80.9% of boys aged 7 to 14 years participate in club sport, the German boys and girls improved the most. These results are consistent with the representative KiGGS study [109], as well as the Danish CHAMPS study [110], where children with club sport background performed much better in almost all PF and MC characteristics investigated. Therefore, in the study of Annesi et al. [111], a more general after-school training program (called YMCA) performed three times per week at lower intensities in 5–12-year olds has already led to significant improvements in body composition, strength, and endurance. Finally, Drenowatz et al. [112] confirmed a significant training effect on PF of 7-year old children when participating in organized club sports just more than once per week. Furthermore, our mediation analysis revealed, at least for running speed, running endurance, and flexibility, that the relevance of club sport training for PF was much higher in German children than in their Chinese counterparts.

In regard to free outside play, which is defined as intrinsically motivated and not provoked by instrumental goal-directed behavior [113], it was shown by Taylor and Kuo [114] that, after extended outside play, even 9 months later, significantly greater improvement in various measures of PF and MC were diagnosed. Thus, it is not surprising that, in our study, outside play activities were also related to the test performances of the children. Similar to club sport training, the availability of nearby outside playgrounds in a middle-sized town is more likely than in a global mega-city such as Shanghai. The environmental factor of outside play contributed to better motor development in the German boys and girls, especially with regard to strength and endurance. This is confirmed by our results that, at least in German boys in the endurance test, better development was seen, maybe due to the larger free space and more open environment that was at hand when free play was executed outside [115].

In regard to the investigated five settings of MVPA, our findings of the cross-cultural comparison of Chinese and German children at the age of 8 to 9 years confirm higher club sport and outdoor play activity levels existing in Germany and slight shortcomings of the Chinese children from the metropolitan cities, e.g., of Shanghai, behind their German counterparts from the mostly more rural counties, at least in power and even more in the endurance ability tested by the 6-min-run. These differences in power and endurance abilities presumably result, at least in part, from a lower volume of total daily physical activities in children of the eastern hemisphere [116], as especially in China, “over-parenting” and “bubble-wrapping” by childcaring grandparents due to the single child policy of the former years and the double-income model of the parents is part of daily routine [100]. Additionally, there is a widespread belief that Chinese children are under strong pressure to perform in school. This pressure, combined with the negative effect of extended learning times that occur in their free time, could partly explain our results. Sun et al. [25], as well as Zhao et al. [26], found that the extracurricular learning activities of Chinese children had a negative impact on leisure time activities. Accordingly, homework and additional knowledge acquisition are placed in the number one and two positions on the priority list of the average Chinese parent so that outdoor play and other MVPAs range far behind. The causes for Chinese strengths, in terms of flexibility and coordinative abilities, may lie partly in genetic factors affecting the body and especially tissue composition [65] or specific Chinese culture features. On the other hand, many modern exercises still bear their roots in Chinese sports history, which is based predominantly on martial arts. Even today, many Chinese people feel closely connected with the old traditions so that martial arts have kept their high status in Chinese culture and are executed frequently by children, adolescents, adults, and also seniors in public spaces, such as parkways. Wang and Olsson [117] discovered that traditional sports were mainly practiced by older people, while the interests of young Chinese people lay primarily in more dynamic leisure sports, such as table tennis, dancing, volleyball, tennis, running, and swimming.

Based on the variety of the sports disciplines played by the German and Chinese children, quite similar PF and MC profiles in Chinese and German elementary school children should be expected. However, the population density in most regions is lower in Germany than in China. This fact, combined with the mostly more rural settings, gives German children numerous inherent play opportunities in the surrounding area. In China, previous research conducted by Zhu et al. [58] has led to inconsistent results as the authors found a better PF (50-m-sprint, sit-and-reach, and sit-ups) in primary school girls (age 6–11 years) from urban environment, but not in boys where a better PF could only be diagnosed in the sit-and-reach test, but not in the 50-m-sprint and sit-up tests. In regard to MC, these authors applied a rope skipping test and found better performances in the boys from urban environment, but not in the girls, who performed on the same level as the girls from the rural site. In studies by Lu et al. [118], performance was found to be superior in urban and metropolitan environments, whereas Zhu et al. [27] did not find differences in children and adolescents living in either rural or urban communities. In Germany, Golle et al. [45] diagnosed children from rural areas to be superior in PF and MC. They also [46] contributed evidence that the children from a rural or at least mixed rural and urban environment show a higher PF and MC than children of urban and especially metropolitan environments. Thus, the comparison of two different large cities certainly provides grounds for criticism. Therefore, it was hypothesized that 8- to 9-year old elementary school children with greater MVPA in particular environments (such as family and leisure sports, school and club sport, and also outside play) would develop better performances in six PF and three MC abilities. As the potential influence of environmental factors, culture, and educational setting on MVPA may vary, there is need of an evidence base for different rural and urban (and metropolitan) districts from both countries. Only when adequate information about MVPA, PF, and MC is at hand can effective policies and programs be established to enhance the activity levels of German, as well as Chinese, children at elementary school age. According to Ferguson et al. [115], there are many factors that can lead to different results, not only the city size. In addition to the different cultural aspects of China and Germany, mental, environmental (suxh as air pollution, accessibility, and size of parkways, etc.) and temporal factors (entrance fees, etc.) may also affect the results. Such factors are difficult to take into account but may also substantially influence motor development during childhood.

Our study has several limitations. The amount of MVPA of the children was assessed by parental reports in a paper format questionnaire, because a direct application in young children might lead to poor reliability and validity [119] if not applied in a child-oriented version [120]. Nevertheless, parental reports may have the general deficit of documented evidence of the psychometric properties in common with other self-report instruments, especially in regard to the risk of overestimation bias [121]. Furthermore, such instruments might provide unreliable data in regard to the comparisons between ethnic groups, as was claimed, for example, by Wang et al. [76] for Chinese youth. Some of the weaknesses of our parental report on the MVPA of their offspring might have resulted from questions being too detailed in the survey. Additionally, many parents could not properly distinguish between family sport, leisure sport, and outdoor playing activities in their answers. For example, due to the fact that, at least in Germany, the majority of the children play outside without supervision, parents may not always know in detail what specific kinds of activity their children perform when going outside to play so that it was unclear to parents how to classify the latter activity as outside play or leisure sport. Furthermore, the distinction between family sport and leisure sport was not trivial. As a consequence, in future research, specific attention has to be paid to further explain the response categories to the parents. An additional study based on observation methods in situ to assess different intensity levels of leisure sport activities in both countries would provide more accurate data, especially on the low-intensity modes of activity such as moderate walking or biking, whereas self-reports seem to be valid primarily in regard to more vigorous running or match play activities of children [122]. Despite the actual lack of nation-wide device-based data on MVPA levels, more reliable and valid values might be attained by controlling MVPA with objective measurement technology (such as pedometry, accelerometry, heart rate control, etc.) rather than with parental estimates, although such technology tends to underestimate the daily MVPA in children due to the typical short bouts of activity at a young age [116].

The main strengths of this study lie in the use of the broadly validated general motor tests in regard to the middle childhood age of 8- to 9-year olds [69,71,72] and in the longitudinal research design that allows for the application of the statistically causal mediation analysis [83], which helps to understand the direct and indirect effects of the MVPA settings in the two countries on particular PF and MC components in 8- to 9-year old scholars in German and Chinese elementary schools. On the one hand, the mostly moderate to large effect sizes of the one-year development of the participants in most of the PF and MC components underlined the appropriateness of the longitudinal study design. In addition, the higher percentage of the explained variance in the regression analyses on the single PF and MC components compared to the regression analyses on the overall PF and MC have led to a more detailed picture of the interrelation of MVPA, PF, and MC in elementary school children from Germany and China.

In summary, this study underlines the benefits of club sports and outside play activities with the aim of improving the PF and MC of boys and girls. Chinese elementary school children might benefit from more intense club sport participation and training, while German boys and girls might similarly benefit from a higher volume of school sports. Additional research is needed to investigate how the development of physical and motor characteristics could be increased in youths in the different MVPA settings. Other possible mediators of the relationship between MVPA, PF, and MC that should be considered in the future are cultural as well as genetic in nature. Flexibility, speed, and strength abilities especially benefit from specific sport disciplines in a different way. Therefore, intervention studies would provide more information regarding the nature of the sport discipline and motor ability relationship. Additionally, the investigated PF and MC characteristics require neuromuscular systems with certain prerequisites, e.g., tissue and muscle fiber composition that are different in Caucasian and Asian children and youth [65]. Additional research is needed to more fully understand the anatomical and physiological mechanisms that may explain the different levels of PF and MC between the two investigated nationalities, especially with regard to condition, coordination, and flexibility.

## 5. Conclusions

This study provided recommendations in both countries to promote PF and MC in elementary school children. To improve PF and MC of children aged 8 to 9 years, sport authorities in China should promote club sport training, whereas German school education authorities should establish a higher volume of school sports. As differences in PF and MC tasks before puberty mostly can be attributed to environmental factors, teachers and coaches are encouraged to use more diversified sports exercises to support PF and MC of participants and to evaluate elementary school curricula in this direction.

This research elucidates the relationship between MVPA, PF, and MC in two different cultures to guide the development of more effective educational strategies and to propose easily accessible fitness devices or playgrounds in parkways to promote MVPA in Chinese and German elementary school children. Toward such national policies and programs to establish an active and healthy lifestyle, international comparison of MVPA, PF, and MC play a crucial role as evidence-based knowledge resources for public health stakeholders.

## Figures and Tables

**Table 1:** Descriptive statistics of physical fitness (PF) and motor competence (MC) variables from the first (Moment 1) and the second assessment (Moment 2).

	**20 m-Sprint (s)**						**Standing Long Jump (cm)**						**Push-Ups (reps)**					
	Moment 1			Moment 2			Moment 1			Moment 2			Moment 1			Moment 2		
	*M*	*SD*	*SE*	*M*	*SD*	*SE*	*M*	*SD*	*SE*	*M*	*SD*	*SE*	*M*	*SD*	*SE*	*M*	*SD*	*SE*
German Boys	4.68	0.35	0.03	4.23	0.37	0.03	128.50	20.00	1.82	138.44	21.22	1.93	13.63	4.61	0.42	16.72	4.48	0.41
Chinese Boys	4.86	0.34	0.03	4.41	0.33	0.03	129.75	18.29	1.54	140.87	17.59	1.47	12.57	3.34	0.28	15.92	3.14	0.26
German Girls	4.75	0.32	0.03	4.30	0.28	0.02	124.56	16.81	1.33	134.52	17.82	1.41	15.16	3.62	0.29	18.09	3.49	0.28
Chinese Girls	5.02	0.35	0.03	4.59	0.34	0.03	125.46	15.17	1.24	134.35	15.43	1.26	12.71	3.72	0.30	15.84	3.06	0.25
Total group	4.83	0.36	0.02	4.39	0.36	0.01	126.92	17.59	0.74	136.88	18.12	0.76	13.56	3.95	0.17	16.67	3.65	0.15
	**Sit-Ups (reps)**						**Bend Forward (cm)**						**6-min-Run (m)**					
	Moment 1			Moment 2			Moment 1			Moment 2			Moment 1			Moment 2		
	*M*	*SD*	*SE*	*M*	*SD*	*SE*	*M*	*SD*	*SE*	*M*	*SD*	*SE*	*M*	*SD*	*SE*	*M*	*SD*	*SE*
German Boys	19.07	4.68	0.43	21.71	5.13	0.47	-2.31	6.59	0.60	0.24	6.62	0.60	905.99	149.47	13.59	991.08	140.31	13.38
Chinese Boys	14.23	6.94	0.58	18.56	7.11	0.59	6.25	5.46	0.46	6.29^ns^	5.70	0.48	824.21	96.95	8.14	862.17	112.40	9.40
German Girls	18.36	5.16	0.41	21.03	4.72	0.37	2.18	6.13	0.48	5.30	6.00	0.47	871.34	112.73	8.94	969.51	101.77	8.39
Chinese Girls	12.33	8.26	0.67	17.05	7.30	0.59	10.18	5.04	0.41	9.92 ^ns^	5.91	0.48	786.89	120.50	6.83	824.21	96.95	7.58
Total group	15.91	7.04	0.29	19.51	6.45	0.27	4.32	7.35	0.31	5.70	6.89	0.29	844.83	119.72	5.00	892.23	125.96	5.37
	**Balancing Backward (steps)**						**Sideward Jumping (reps)**						**Ball Throw (m)**					
	Moment 1			Moment 2			Moment 1			Moment 2			Moment 1			Moment 2		
	*M*	*SD*	*SE*	*M*	*SD*	*SE*	*M*	*SD*	*SE*	*M*	*SD*	*SE*	*M*	*SD*	*SE*	*M*	*SD*	*SE*
German Boys	26.24	9.18	0.83	29.47	9.60	0.87	23.95	6.16	0.56	27.91	7.41	0.67	13.79	3.80	0.35	16.27	4.32	0.39
Chinese Boys	23.17	9.59	0.80	27.47	8.52	0.71	25.25	6.05	0.51	28.92	6.61	0.55	10.50	2.97	0.25	12.22	3.17	0.26
German Girls	30.89	8.71	0.69	35.48	7.88	0.62	27.08	5.20	0.41	29.70	5.79	0.46	9.41	2.51	0.20	11.44	2.88	0.23
Chinese Girls	25.94	10.14	0.83	30.77	8.66	0.71	25.87	5.63	0.46	29.78	5.17	0.42	8.21	2.22	0.18	9.61	2.16	0.18
Total group	26.70	9.82	0.41	30.99	9.12	0.38	25.65	5.83	0.24	29.15	6.25	0.26	10.29	3.49	0.15	12.16	3.91	0.16

Legend: Other than the bend forward test in Chinese boys and girls, all differences between Moment 1 and Moment 2 are significant (*p* < 0.05).

**Table 2:** Statistics of the one-year development (delta) of all physical fitness (PF) and motor competence (MC) variables between the first (Moment 1) and the second assessment (Moment 2).

	**20 m-Sprint (s)**					**Standing Long Jump (m)**					**Push-Ups (reps)**				
	**Delta**					**Delta**					**Delta**				
	*M*	*SD*	*d*	*T_GER-CHN;569_*	*p*	*M*	*SD*	*d*	*T_GER-CHN;570_*	*p*	*M*	*SD*	*d*	*T_GER-CHN;570_*	*p*
Chinese children	0.44	0.30	1.46	0.33	0.74	10.06	13.26	0.79	−0.17	0.99	3.24	4.00	0.81	−0.73	0.47
German children	0.45	0.19	2.36			10.04	13.05	0.79			3.00	3.75	0.80		
Total group	0.45	0.26	1.73			10.05	13.14	0.76			3.12	3.88	0.80		
	**Sit-Ups (reps)**					**Bend Forward (cm)**					**6-min-Run (m)**				
	**Delta**					**Delta**					**Delta**				
	*M*	*SD*	*d*	*T_GER-CHN;569_*	*p*	*M*	*SD*	*d*	*T_1;569_*	*p*	*M*	*SD*	*d*	*T_GER-CHN;570_*	*p*
Chinese children	4.56	6.16	0.74	−4.31	0.01	−0.17	4.22	0.12	8.58	0.01	39.23	98.86	0.39	2.40	0.02
German children	2.66	4.22	0.63			2.87	4.23	0.67			60.76	109.55	0.55		
Total group	3.62	5.24	0.69			1.33	4.49	0.29			49.30	104.46	0.47		
	**Balancing Backward (steps)**					**Sideward Jumping (reps)**					**Ball Throw (m)**				
	**Delta**					**Delta**					**Delta**				
	*M*	*SD*	*d*	*T_GER-CHN;570_*	*p*	*M*	*SD*	*d*	*T_GER-CHN;568_*	*p*	*M*	*SD*	*d*	*T_GER-CHN;545_*	*p*
Chinese children	4.71	8.75	0.53	−0.97	0.33	3.85	5.31	0.72	−1.48	0.14	1.57	2.21	0.71	3.47	0.01
German children	4.01	8.67	0.46			3.20	5.15	0.62			2.34	2.37	0.98		
Total group	4.37	8.71	0.50			3.53	5.24	0.67			1.90	2.31	0.82		

**Table 3:** Descriptive statistics of the MVPA executed in five different settings over a one-year period in German and Chinese boys and girls.

**Family Sport**					
	***M*^1^**	***SD***	***d***	***T_GER-CHN;208_*** ***T_GER-CHN;237_***	***p***
German Boys	1.00	0.68	0.21	1.52	0.13
Chinese Boys	0.84	0.76			
German Girls	0.76	0.66	0.04	−0.33	0.75
Chinese Girls	0.79	0.81			
**School Sport**					
	***M*^1^**	***SD***	***d***	***T_GER-CHN;206_*** ***T_GER-CHN;231_***	***p***
German Boys	2.41	0.73	0.17	−1.23	0.22
Chinese Boys	2.61	1.29			
German Girls	2.35	0.74	0.28	−2.38	0.02
Chinese Girls	2.68	1.38			
**Outside Play Activities ^2^**					
	***M*^1^**	***SD***	***d***	***T_GER-CHN;205_*** ***T_GER-CHN;231_***	***p***
German Boys	4.12	2.53	1.14	7.90	0.01
Chinese Boys	1.65	1.26			
German Girls	3.23	2.18	1.38	6.61	0.01
Chinese Girls	1.58	1.35			
**Leisure Sport**					
	***M*^1^**	***SD***	***d***	***T_GER-CHN;182_*** ***T_GER-CHN;214_***	***p***
German Boys	0.66	0.83	0.21	−1.43	0.15
Chinese Boys	0.85	0.96			
German Girls	0.76	0.84	0.10	−0.78	0.44
Chinese Girls	0.85	0.84			
**Club Sport**					
	***M*^1^**	***SD***	***d***	***T_GER-CHN;210_*** ***T_GER-CHN;234_***	***p***
German Boys	1.13	0.75	0.76	5.74	0.01
Chinese Boys	0.44	0.88			
German Girls	0.90	0.72	0.52	4.21	0.01
Chinese Girls	0.46	0.88			

Legend: M = mean; SD = standard deviation; *d* = Cohen’s d; T = Test statistic of the difference between German and Chinese children; *p* = significance. ^1^ Units are the numbers of days of moderate-to-vigorous physical activities (MVPA) per week. ^2^ In the “outside play” category, only days were recorded when children had played outside for more than 60 min [13].

## Data Availability

The data associated with the study are not publicly available but are available from the corresponding author on reasonable request.
